# Synchronous Bilateral Breast Invasive Ductal Carcinoma With Osteoclast-Like Stromal Giant Cells in a 44-Year-Old Woman: A Case Report

**DOI:** 10.7759/cureus.105310

**Published:** 2026-03-16

**Authors:** Hinako Tezuka, Akira Matsui, Yuya Murata, Erina Odani, Emi Tsukiyama, Marika Sato, Manami Sasahara, Hirohito Seki, Takayuki Kinoshita

**Affiliations:** 1 Department of Breast Surgery, NHO Tokyo Medical Center, Tokyo, JPN; 2 Department of Pathology, NHO Tokyo Medical Center, Tokyo, JPN; 3 Department of Breast Surgery, NHO Tokyo Medical center, Tokyo, JPN

**Keywords:** brca 1/2, ductal carcinoma in situ (dcis), invasive ductal carcinoma of the breast, osteoclast-like giant cells, osteoclast-like stromal giant cell, synchronous bilateral breast cancer, tumor micro environment

## Abstract

Breast carcinoma with osteoclast-like stromal giant cells (OCGC) is a rare histological variant of invasive breast carcinoma. While it typically presents as a unilateral disease, its clinicopathological significance and the mechanisms underlying its formation remain incompletely understood. To our knowledge, a case of synchronous bilateral primary invasive ductal carcinoma with OCGCs has not been previously reported.

A 44-year-old premenopausal woman was diagnosed with synchronous bilateral invasive ductal carcinoma with OCGCs. Imaging revealed small masses in both breasts, and core needle biopsies demonstrated invasive carcinoma with associated non-invasive components and numerous OCGCs in the tumor stroma. Both tumors were hormone receptor-positive and human epidermal growth factor receptor 2 (HER2)-negative (luminal A-like). The patient underwent bilateral mastectomy and sentinel lymph node biopsy, which showed no lymph node metastasis. The postoperative course was uneventful, and the patient has remained recurrence-free for six months after surgery. Notably, final pathological examination confirmed independent ductal carcinoma in situ (DCIS) components with OCGCs in both breasts, supporting the diagnosis of bilateral primary tumors rather than metastatic disease. Immunohistochemically, the OCGCs were CD68-positive, confirming their macrophage lineage. Genetic testing showed no pathogenic *BRCA1/2* variants.

This report highlights an extremely rare, pathologically confirmed case of synchronous bilateral primary breast carcinoma with OCGCs. The presence of OCGCs in both invasive and in situ components suggests that their formation can be induced at early stages of tumor development, likely reflecting a distinct immune-reactive tumor microenvironment driven by host-related factors.

## Introduction

Breast carcinoma with osteoclast-like stromal giant cells (OCGC) is a rare variant of invasive breast carcinoma and has been classified as a special histological type [1,2]. Its reported incidence ranges from approximately 0.05% to 2.0% of all breast cancers [3,4]. This entity is characterized by the presence of numerous osteoclast-like giant cells within the tumor stroma [1]. Despite its distinctive histological features, the clinicopathological significance and underlying mechanisms of tumorigenesis remain not fully understood. Breast carcinoma with OCGC is typically reported as a unilateral disease, and to the best of our knowledge, no cases of synchronous bilateral breast carcinoma with OCGC have been previously reported in the literature. We report an extremely rare case of synchronous bilateral invasive breast carcinoma with OCGC. We present the clinicopathological features of this case and discuss the possible mechanisms of tumor development, with particular emphasis on the tumor microenvironment, based on a review of the relevant literature.

## Case presentation

A 44-year-old Japanese premenopausal woman (gravida 1, para 1) presented with left breast pain. She had a history of ovarian endometriotic cysts and was being treated with a levonorgestrel-releasing intrauterine system (LNG-IUS) at the time of breast cancer diagnosis. There was no remarkable family history of breast or ovarian cancer. She had visited a local clinic for evaluation, where breast ultrasonography had revealed a mass in the right breast, and she had been referred to our institution within about one week for further evaluation.

**Figure 1:**
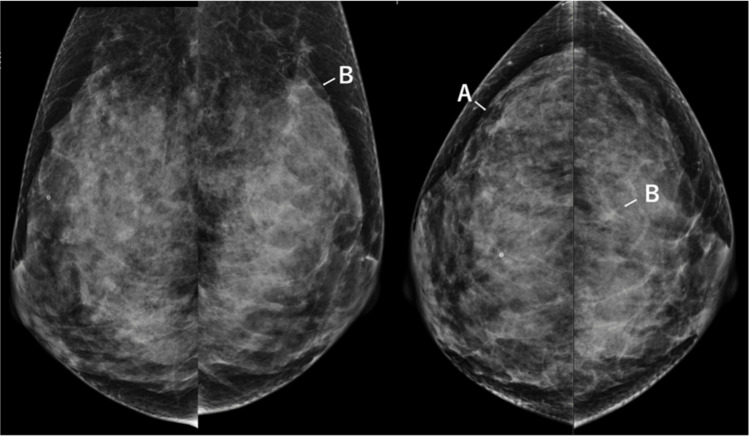
Mammographic findings (A) Focal asymmetric density (FAD) with architectural distortion is observed in the medial portion of the right breast. (B) Focal asymmetric density (FAD) with architectural distortion is observed in the upper central area of the left breast

**Figure 2:**
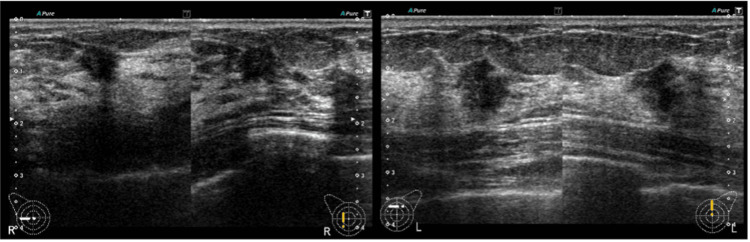
Breast ultrasonographic findings An irregular hypoechoic mass measuring approximately 8 mm in maximum diameter is observed at the 9 o’clock position in the right breast, and an irregular hypoechoic mass measuring approximately 11 mm in maximum diameter is observed at the 12 o’clock position in the left breast

Mammography demonstrated focal asymmetric density with architectural distortion in the medial portion of the right breast and the upper central area of the left breast (Figure 1). Breast ultrasonography revealed hypoechoic masses measuring 8 mm at the 9 o’clock position in the right breast and 11 mm at the 12 o’clock position in the left breast (Figure 2).

**Figure 3:**
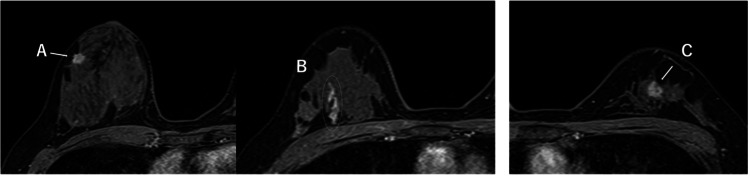
Contrast-enhanced MRI findings (A) An irregularly enhancing mass is observed at the 9 o’clock position in the right breast. (B) Non-mass enhancement is observed at the 11 o’clock position in the right breast. (C) An irregularly enhancing mass is observed at the 12 o’clock position in the left breast MRI: magnetic resonance imaging

Ultrasound-guided core needle biopsies were performed for both lesions. Histopathological examination revealed invasive breast carcinoma with an associated non-invasive component in both breasts. Numerous osteoclast-like giant cells were observed within the tumor stroma, leading to a diagnosis of synchronous bilateral breast carcinoma with OCGC. Immunohistochemical analysis showed that both tumors were hormone receptor-positive and human epidermal growth factor receptor 2 (HER2)-negative, corresponding to the luminal A subtype. Preoperative breast MRI demonstrated additional non-mass enhancement at the 11 o’clock position in the right breast, separate from the biopsied lesion (Figure 3). Contrast-enhanced CT for preoperative staging revealed no distant metastasis.

After completion of the diagnostic evaluation, surgery was performed approximately one month later. The patient underwent bilateral mastectomy with bilateral sentinel lymph node biopsy. No lymph node metastases were identified. On final pathological examination, the right breast contained a 14-mm invasive ductal carcinoma with OCGC corresponding to the biopsied lesion, which showed high nuclear grade (grade 3) and a relatively high Ki-67 labeling index of 30%. In addition, a separate lesion corresponding to the area of non-mass enhancement on MRI revealed ductal carcinoma in situ (DCIS) with OCGC, measuring 35 mm in extent, distinct from the invasive carcinoma.

**Figure 4:**
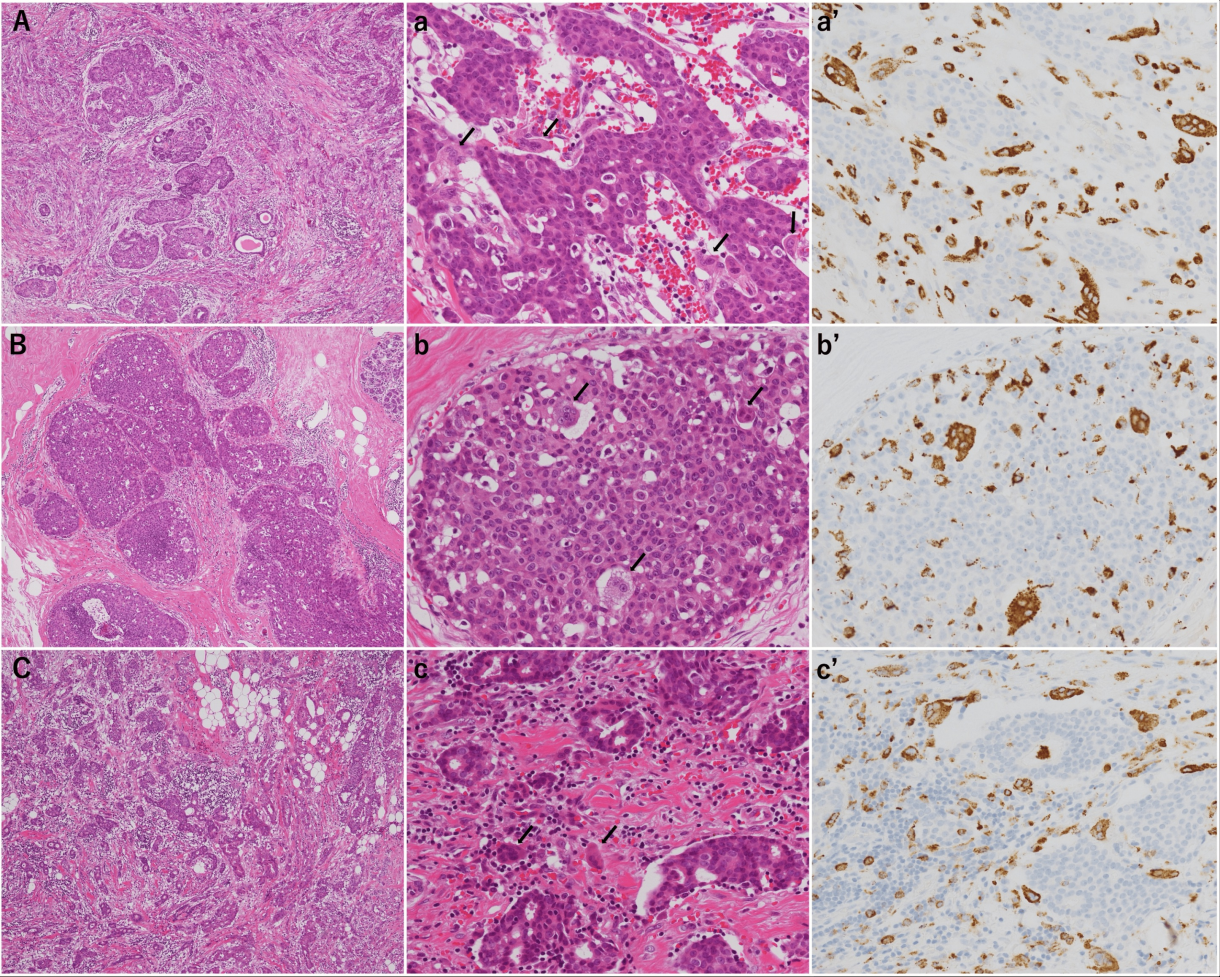
Histopathological and immunohistochemical findings of the breast lesions The figure displays a side-by-side comparison of the three lesions. The left column (A, B, and C) shows low-power views (×40, hematoxylin and eosin (HE) staining). The middle column (a, b, and c) shows high-power views (×200, HE staining), where black arrows indicate osteoclast-like giant cells (OCGCs). The right column (a', b', and c') shows CD68 immunohistochemical staining of the corresponding areas (×200) (A, a, a') Right breast lesion at the 9 o’clock position: invasive ductal carcinoma accompanied by numerous OCGCs surrounding the tumor cells. The OCGCs show strong CD68 positivity (a') (B, b, b') Right breast lesion at the 11 o’clock position: ductal carcinoma in situ (DCIS) with OCGCs within the ductal lumen and partially surrounding the tumor cells. The OCGCs show strong CD68 positivity (b') (C, c, c') Left breast lesion at the 12 o’clock position: invasive ductal carcinoma accompanied by numerous OCGCs surrounding the tumor cells. The OCGCs show strong CD68 positivity (c')

In contrast, the left breast harbored a 15-mm invasive ductal carcinoma with OCGC, with low nuclear grade (grade 1) and a low Ki-67 labeling index of 10%. Based on these findings, the final pathological staging was pT1cN0M0, pStage IA synchronous bilateral breast carcinoma. Immunohistochemical staining for CD68 demonstrated positivity in the osteoclast-like giant cells in all lesions, confirming their macrophage lineage (Figure 4). The clinicopathological characteristics of the bilateral tumors, including invasive and in situ lesions, are summarized in Table 1.

**Table 1:** Clinicopathological comparison of bilateral breast tumors IDC: invasive ductal carcinoma; DCIS: ductal carcinoma in situ; ER: estrogen receptor; PgR: progesterone receptor; HER2: human epidermal growth factor receptor 2

Parameter	Right IDC	Right DCIS	Left IDC
Tumor size	13 × 10 mm	35 × 30 mm	15 × 12 mm
Nuclear grade	3	2	1
Ki-67 index	30%	10%	10%
ER	Positive	Positive	Positive
PgR	Positive	Positive	Positive
HER2 (IHC)	2+	2+	1+
DCIS component	Present	—	Present

Genetic testing revealed no pathogenic variants in *BRCA1 *or *BRCA2*. The Oncotype DX recurrence score, evaluated for the lesion at the 9 o’clock position in the right breast, was 13. As adjuvant therapy, the patient was started on oral S-1 chemotherapy in combination with endocrine therapy, which has been continued for six months postoperatively without any evidence of recurrence.

## Discussion

Breast carcinoma with OCGC is a rare histological variant; however, its pathogenesis and prognostic significance remain a subject of debate [1,2,3]. OCGCs are generally considered reactive multinucleated giant cells rather than neoplastic components, likely originating from tumor-associated macrophages (TAMs) recruited to the tumor stroma. In line with previous reports, the CD68 positivity observed in our case supports the hypothesis that these cells arise from the fusion of mononuclear macrophage-lineage cells [1,4].

The most remarkable feature of the present case is the synchronous bilateral occurrence of invasive ductal carcinoma with OCGCs. To date, only a single bilateral case of breast carcinoma with osteoclast-like giant cells has been reported [5]; however, it involved invasive lobular carcinoma, and the possibility of contralateral metastasis could not be definitively excluded. In contrast, the present case exhibited not only invasive carcinoma but also independent DCIS components accompanied by OCGCs in both breasts. This finding provides compelling pathological evidence that the tumors represented separate primary malignancies rather than metastatic disease. To our knowledge, this is the first report to pathologically confirm synchronous bilateral primary invasive ductal carcinoma with OCGCs.

While bilateral breast cancer often suggests a genetic predisposition, no pathogenic variants in *BRCA1* or *BRCA2 *were identified in this patient, and the molecular subtypes were concordant. This suggests that the bilateral development may be driven by shared host-related factors, such as systemic immune or inflammatory responses, rather than localized stochastic events alone. Furthermore, the presence of OCGCs within the DCIS components indicates that OCGC formation is not exclusive to the invasive phase but can be induced during early-stage tumorigenesis. The RANK/RANKL signaling pathway has been implicated in this recruitment and fusion of macrophages [1,2,6]. Although RANKL expression was not evaluated here, similar immunological crosstalk within the microenvironment likely played a key role.

Against this background of an immune cell-rich tumor microenvironment, it can be theoretically postulated that immune checkpoint inhibitors may represent a potential therapeutic option [1]. However, to date, there have been no clinical trials or substantial case series specifically evaluating the efficacy of immune checkpoint inhibitors in breast carcinoma with OCGCs, and thus, evidence regarding their therapeutic benefit remains unclear. Although the use of immune checkpoint inhibitors in breast carcinoma with OCGCs may be biologically plausible, the current clinical evidence is insufficient to support their inclusion as a standard treatment. Further investigation, including the accumulation of additional cases and comprehensive molecular and pathological analyses, is warranted.

Breast carcinoma most commonly metastasizes to the bone, lung, liver, and brain. A specific metastatic pattern has not been established for breast carcinoma with OCGCs. However, the biological behavior of this rare variant is considered to reflect the characteristics of the underlying histological subtype, and distant metastases to various organs may occur [1,2,4]. Consistent with this, previously reported cases have demonstrated metastases to common sites such as bone, lung, and liver.

Regarding prognosis, several studies suggest a relatively favorable outcome for breast carcinoma with OCGCs [4,7], potentially due to robust immune cell infiltration and low rates of lymph node metastasis. However, recent evidence indicates that prognosis is determined more by the underlying tumor biology (e.g., nuclear grade, Ki-67) than by the presence of OCGCs themselves [8,9,10]. In our case, the right breast showed a higher grade and Ki-67 index, necessitating a more cautious follow-up despite the generally favorable subtype.

In conclusion, synchronous bilateral breast carcinoma with OCGCs is an exceptionally rare clinical entity. This report underscores the importance of the tumor microenvironment and host-related immunological factors in the pathogenesis of this rare variant.

## Conclusions

We presented an exceptionally rare case of synchronous bilateral breast carcinoma with osteoclast-like stromal giant cells. Our findings suggest that the formation of OCGCs is closely linked to tumor-mediated immune responses and the specific tumor microenvironment. Further accumulation of similar cases, alongside comprehensive molecular and immunopathological analyses, is essential to fully elucidate the biological behavior and clinical significance of this rare breast cancer variant.
